# Dissecting Fibroblast Heterogeneity in Health and Fibrotic Disease

**DOI:** 10.1007/s11926-020-00903-w

**Published:** 2020-06-19

**Authors:** Tanya J Shaw, Emanuel Rognoni

**Affiliations:** 1 Centre for Inflammation Biology & Cancer Immunology, Department of Inflammation Biology, School of Immunology & Microbial Sciences, New Hunt’s House, Guy’s Campus, King’s College London, London, SE1 1UL UK; 2 Centre for Endocrinology, William Harvey Research Institute, Barts and the London School of Medicine and Dentistry, Queen Mary University of London, London, EC1M 6BQ UK

**Keywords:** Fibroblast, Cell heterogeneity, Single-cell transcriptomics, Fibrosis, Tissue injury, Myofibroblast

## Abstract

**Purpose of Review:**

Fibroblasts, the major cell population in all connective tissues, are best known for their role in depositing and maintaining the extracellular matrix. Recently, numerous specialised functions have been discovered revealing unpredicted fibroblast heterogeneity. We will discuss this heterogeneity, from its origins in development to alterations in fibrotic disease conditions.

**Recent Findings:**

Advances in lineage tracing and single-cell transcriptional profiling techniques have revealed impressive diversity amongst fibroblasts in a range of organ systems including the skin, lung, kidney and heart. However, there are major challenges in assimilating the findings and understanding their functional significance. Certain fibroblast subsets can make specific contributions to healthy tissue functioning and to fibrotic disease processes; thus, therapeutic manipulation of particular subsets could be clinically beneficial.

**Summary:**

Here we propose that four key variables determine a fibroblast’s phenotype underpinning their enormous heterogeneity: tissue status, regional features, microenvironment and cell state. We review these in different organ systems, highlighting the importance of understanding the divergent fibroblast properties and underlying mechanisms in tissue fibrosis.

## Introduction

Fibroblasts, the main cell population in connective tissues, have a reputation for their important structural role of extracellular matrix (ECM) deposition and remodelling, but are also appreciated to make important contributions to numerous vital biological processes including wound repair, immune responses and differentiation into other cell types, whilst contributing to fibrotic disease and tumour development. In spite of their impressive range of functions, a precise definition of a fibroblast remains elusive, perhaps because of the enormous heterogeneity within this cell population. Following a brief introduction to generalised features of fibroblasts, this review will discuss recent advances in dissecting the diversity of fibroblasts in different organ systems (skin, lung, liver, kidney and heart), aiming to highlight the functional significance of different subpopulations in tissue repair and fibrosis.

## General Fibroblast Features

Before delving into the divergent features of newly described fibroblast lineages or subpopulations, it is important to consider some of their conventional properties. Fibroblasts are migratory and highly proliferative during development; then, in most adult tissues, they quiesce and persist long term in homeostasis. These fibroblasts may appear in a passive state, however, they are highly metabolically active. They continuously deposit and remodel their surrounding ECM, and patrol the composition and mechanical properties of their environment. Also, an ability to modulate the immune response by expressing different cytokines is a common feature.

Upon tissue damage, fibroblasts can quickly exit their quiescent state and become “activated” in response to a plethora of cues. Depending on the cues, cells may start to proliferate, migrate to the injury site, differentiate into highly contractile myofibroblasts and/or increase ECM deposition and remodelling. Ageing and/or environmental stressors (e.g. DNA damage, oxidative stress) can also influence fibroblast biology, potentially promoting senescence [1].

Fibroblasts are notoriously plastic, as demonstrated by the fact that they are routinely reprogrammed into inducible pluripotent stem cells [2]. Differentiation into contractile myofibroblasts is one in situ example of their cellular plasticity [3], but they also have the ability to specialise in different tissues, becoming adipocytes and dermal papilla cells or even cartilage-like cells [4] in the skin, or smooth muscle cells in the lung airway compartment [5]. Interestingly, there are also numerous scenarios in which fibroblasts lack plasticity. For example, fibroblasts derived from diseased tissue, such as chronic wounds, fibrosis or cancer, have a persistent pathological phenotype [6, 7], which likely has an epigenetic basis [8]. Similarly, transplantation experiments of gingival fibroblasts into dorsal skin indicate that they retain aspects of their original identity, including a relatively non-fibrotic phenotype [9].

Close histological examination of most tissue types shows a non-uniform stromal compartment (e.g. with variable cell and matrix density and composition) and indeed the morphology and behaviour of the fibroblasts isolated from different regions within a tissue are also variable [10–15]. Advances in lineage tracing and transcriptional profiling techniques are enabling us to better understand the diversity of these fascinating cells, although there are challenges in assimilating the increasingly abundant data and making sense of it all.

## A Strategy for Fibroblast Stratification

**Fig. 1:**
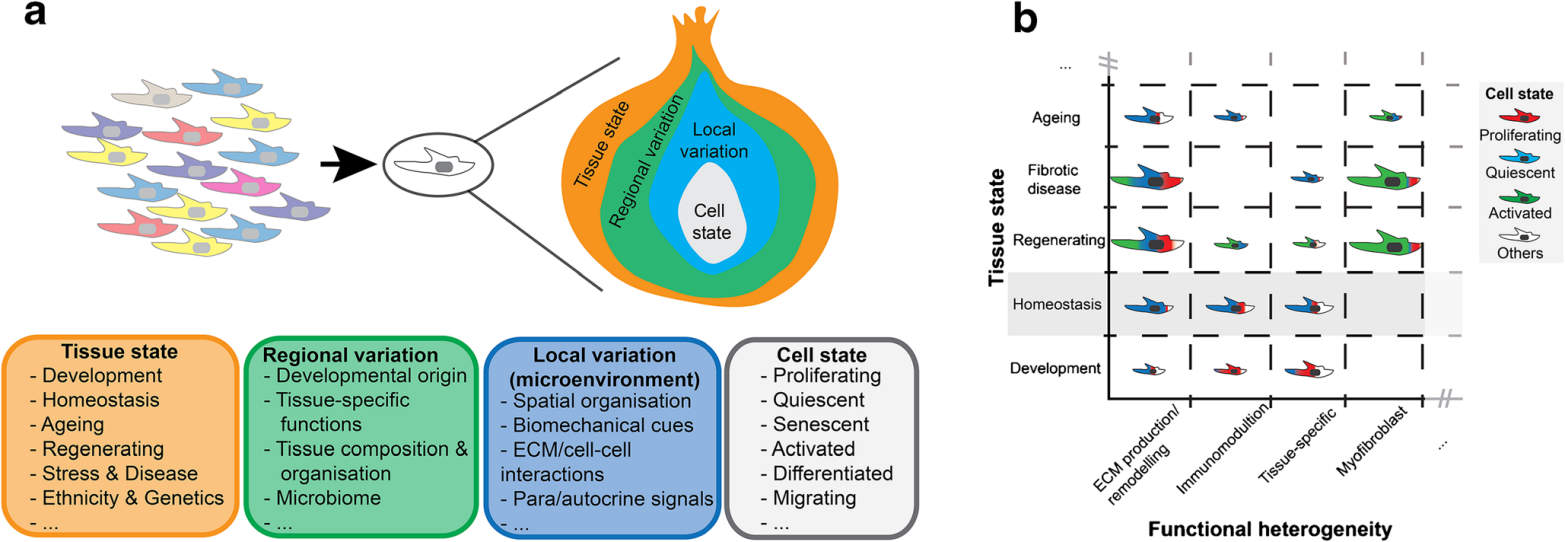
Strategies for fibroblast stratification. **a** Layers of the fibroblast phenotype. Within a tissue, the diversity of the fibroblast population (e.g. as identified by single-cell RNA-seq) will be the combined reflection of the tissue state, regional/anatomical variations, local heterogeneity (microenvironment) and cellular state. **b** Discovering how different tissue states influence fibroblast heterogeneity. Single-cell RNA-seq generally starts with adult homeostatic tissue to define fibroblast heterogeneity at a local level (e.g. tissue biopsy; highlighted in grey). These efforts have revealed subpopulations with distinct functionality that are predominately in a quiescent state. How these lineages develop is variable between organ systems, but involves significant proliferation of a pool of multipotent progenitors that ultimately differentiate into specialised subsets. Upon tissue injury, fibroblasts become activated and may transiently change their relative abundances and functionality. New subpopulations (e.g. myofibroblast) may appear during this process from one or several precursors. More significant and persistent changes in fibroblast heterogeneity have been observed in fibrotic disease conditions (e.g. accumulation and dominance of continuously active myofibroblasts, which themselves are diverse). With age, fibroblast abundance and diversity decline, which may impair organ function or ability to regenerate. *Cell size indicates fibroblast subpopulation abundance and the colouring illustrates different cellular states within a functionally distinct population. schematic provides some illustrative examples only that is based upon current literature but do not represent a specific organ system.*

We propose that the transcriptomic signature and in turn the phenotype of any one fibroblast is the product of four layers of influence (Fig. 1a). First, is the *tissue condition/state*, which describes if the tissue is developing, homeostatic, ageing, regenerating, stressed or diseased. This is followed by a *regional or anatomical heterogeneity*, which can vary in terms of tissue composition (e.g. vascularisation, innervation, abundance of fat/muscle), developmental origin (e.g. mesoderm/neural crest in the skin, epicardium/endothelium in the heart), microbiome and the requirement for tissue-specific functions (e.g. supporting hair follicle formation in the skin or bone resorbtion in the synovium). The third layer is the *local heterogeneity*, which reflects the immediate microenvironment of the fibroblast, including the ECM, cell and matrix interactions, paracrine and autocrine signals and biomechanical cues (e.g. tissue stiffness, shear force). Lastly, the *cell phenotype* reflects its state, such as quiescence, proliferation, senescence, activation, migration or differentiation. Considering all of these variables, it is perhaps not surprising that single-cell RNA sequencing (RNA-seq) datasets have revealed extraordinary heterogeneity amongst fibroblast populations in all tissue types and organ systems. The challenge now is to consider how these transcriptionally defined clusters differ with respect to functionality, likely making specific contributions to healthy tissue homeostasis and disease processes (Fig. 1b). How this appears in different organ systems will now be considered.

## Fibroblast Heterogeneity in Development, Homeostasis and Fibrosis

### Skin

The skin dermis develops from multipotent fibroblasts, which clonal lineage tracing experiments have revealed are highly proliferative during embryonic development, but after birth, they switch to a quiescent state efficient at ECM deposition [16]. The cells then differentiate into spatially distinct lineages, creating the dermal sublayers: papillary, reticular and dermal white adipose tissue (DWAT) [17]. Combining computational modelling with lineage tracing suggests that the organisation of different lineages is controlled by a balance of cell proliferation and ECM deposition [18•]. Lineage tracing and transcriptomic analyses of mouse skin in homeostasis have provided insight into the functional variations between cells in the different layers at single anatomical sites [9, 16, 19, 20, 21•]. Briefly, papillary fibroblasts, beneath the basement membrane, have an active Wnt signalling signature and are required for hair follicle formation, giving rise to arrector pili muscle, dermal papilla and dermal sheath cells. Fibroblasts in the reticular dermal layer highly express genes associated with ECM and immune signalling and give rise to lipid-filled adipocytes of the DWAT.

A comparable local diversity is apparent in human skin at rest; one single-cell RNA-seq analysis identified two major fibroblast lineages (characterised by co-expression of SFRP2 and DPP4 or FMO1 and LSP1), which could be further subdivided into several additional subpopulations [22•]. A similar study distinguished five mesenchymal populations, which were described as upper and lower dermal fibroblasts, pericytes and two uncharacterised populations [23•]. Comparing young and old human skin in an extensive RNA-seq analysis Solé-Boldo et al., identified four major dermal fibroblast populations with functionally distinct transcriptomic signatures and spatial distribution and defined these as secretory-reticular, secretory-papillary, pro-inflammatory and mesenchymal fibroblasts [24]. Notably, another RNA-seq study mapping six distinct fibroblast populations in human skin failed to correlate established human papillary and reticular fibroblast markers to any specific clusters [25]. This perhaps indicates that the fibroblast transcriptome is strongly influenced by other factors, such as tissue state and biopsy location (Fig. 1a).

The importance of tissue state was clearly demonstrated by a single cell RNA-seq study of mouse skin during hair growth uncovering a transient switch in the transcriptional signature in two of the four identified dermal fibroblast population upon hair growth induction [21•]. The variations in skin from different anatomical sites include dermal thickness, abundance of fat, vascularity, nerve density, hair follicle density, and immune cell composition. The dermis also has distinct developmental origins, depending on the site, with the craniofacial skin uniquely developing from the neural crest cells, whereas the majority of the body arises from the mesoderm (reviewed in [26]). All of these variable regional features are anticipated to influence the local, functional heterogeneity being uncovered.

The distinct functionality of the different skin fibroblast lineages really surface when considered during wound repair (Fig. 1b). When wounded, all fibroblasts in the surrounding dermis are exposed to numerous stimuli triggering their “activation”, such as serum and significant mechanical changes [27]. However, fibroblast subpopulations differ in their wound healing response. For example, lineage-tracing studies in mouse skin wounds demonstrated that cells in the lower dermis are the first to repopulate the damaged tissue and mediate the ECM deposition, whereas papillary fibroblasts move in later and may have a role in remodelling and/or appendage regeneration [18•, 19]. Adipocytes and fascial fibroblasts are also thought to significantly contribute to wound repair [28, 29], but likely not dermal papilla or arrector pili muscle cells [30]. Additionally, a perivascular myofibroblast progenitor with pericyte characteristics and high expression of ADAM12 (a disintegrin and metalloprotease 12) has been identified as an important mediator of scarring [31]; however, to what extent pericytes directly contribute to tissue repair beyond angiogenesis is still controversial. The heterogeneity of scar-forming cells within mouse skin wounds has been further dissected by Guerrero-Juarez et al.; their RNA-seq analysis identified 12 activated fibroblast clusters, including a rare myeloid-derived cell population [32•]. These clusters not only differed in transcription factor and receptor-ligand expression but also in signatures of signalling pathway activation, their cell cycle state and spatial distribution. Consistent with these signatures, it has been discovered that only selective cell populations respond to certain paracrine signals; for example, a specific macrophage subset specifically promotes the proliferation of an activated adipocyte precursor population [33•]. Although the functional significance of this cellular diversity in a wound bed requires further study, it supports the idea that there are multiple activated fibroblast states.

The influence of tissue age on skin fibroblast heterogeneity and phenotype has also been addressed; transcriptional profiling and flow cytometry have revealed that age influences, and indeed decreases, heterogeneity in homeostasis and wound repair [16, 24, 33•, 34]. This may be attributable to changes in the local microenvironment (ECM, cellular and paracrine signals), cell intrinsic factors and systemic metabolism. Heterogeneity may equally be reduced in skin fibrosis, with specific subpopulations committed to a particular state of activation or differentiation dominating the ongoing tissue repair process. In an irradiation-induced skin fibrosis model, a CD26-positive fibroblast subset developmentally derived from an *Engrailed*-positive cell population is mainly responsible for the fibrotic ECM deposition, and depletion using a diphtheria toxin strategy improved the phenotype [9]. A similar result was achieved by selectively deleting perivascular pro-fibrotic ADAM12+ progenitors [31]. Thus, targeting specific fibroblast subpopulations has the promise to suppress fibrosis development without affecting regenerative fibroblast populations.

At the conclusion of a healthy repair process, although there is inevitably a residual scar, there is in fact the potential for significant resolution of the scar myofibroblasts. How their fate is determined and regulated remains largely unclear, but apoptosis and senescence are common outcomes. We do not yet know whether different fibroblast populations have varying propensities to apoptose or senesce, or even if they maintain distinct properties in the senescent state; certainly, some have relevant pro-fibrotic properties [35•]. Further differentiation events are also possible; for example, myofibroblasts stimulated by BMP signalling from regenerating hair follicles can convert to adipocytes and replenish the DWAT [36], and myofibroblasts influenced by epidermal hedgehog signalling can adopt a dermal papilla fate, which in turn is able to stimulate hair follicle neogenesis [37]. It will be interesting to dissect if myofibroblasts retain a memory of their original lineage identity, and whether all myofibroblasts are equally able to convert into specific cell fates when exposed to a particular microenvironment. Also, our current understanding about fibroblast heterogeneity in skin fibrosis is only being inferred from the wound-associated scarring process, but ideally, future work profiling of fibroblast subsets in mouse models of skin disease and human lesions will be informative about differences in manageable versus pathological fibrosis.

### Lung

Connective tissue in the lung includes a diverse collection of mesenchymal cell populations: airway smooth muscle, vascular smooth muscle, pericytes surrounding the abundant blood vessels and fibroblasts. To dissect mesenchymal cell heterogeneity in lung homeostasis, Zepp et al. combined histological analysis of cell signalling reporters for PDGFRα, Wnt2 and Axin2 with single-cell RNA-seq, ultimately stratifying the cell populations spatially and functionally [38••]. Five key subpopulations were identified transcriptionally, which nicely overlapped with the reporters used, but the spatial distribution really helped illuminate their distinct functions. PDGFRα+ cells were generally found in the alveolar niche, versus PDGFRα− in the vicinity of airways and blood vessels. This binary grouping could be further subdivided; PDGFRα+/Axin2+ cells (designated “MANCs”, mesenchymal alveolar niche cells) were particularly important for alveolar epithelial cell growth and self-renewal, whereas PDGFRα−/Axin2+ defined a myofibrogenic progenitor population, which were major contributors to pathogenic myofibroblasts after injury (described below). With a similar approach, Lee et al. revealed that Lgr5 and Lgr6 expression defines two spatially and functionally distinct populations in the healthy mouse lung mesenchyme [39]. Comparably with Zepp et al., a population located in the alveolar niche emerged (which here was Lgr5+), as well as an Lgr6+ smooth muscle cell subpopulation in the airway compartment. The authors considered their Lgr5+ cells partially overlapping with the PDGFRα+/Axin2+ MANCs described above, and the Lgr6-expressing smooth muscle cell subpopulation is anticipated to be a progeny of Axin2+ progenitors close to the airways. Adding to the cellular complexity, lipofibroblasts are another distinct cell population detected by Xie et al. in their single-cell RNA-seq analysis of healthy mouse lung tissue [40]. These are lipid-containing interstitial fibroblasts anticipated to be important for alveolar development and regeneration [41].

It is not yet clear how the subpopulations identified in mouse will map onto the human lung; however, single-cell analysis on healthy human lungs has identified two major (SPINT2 high and MFAP5 high) and one minor (WIF1 high) groupings [42••]. Divergent transcriptional profiles, including variable expression of ECM genes, infer distinct functionality that may correlate with MANCs or airway smooth muscle cells described in mouse, but additional localisation and functional assays are needed. There was no evidence of lipofibroblasts in this study, suggesting that this cell designation may not be detected in normal human lungs, or at least in the region biopsied.

Lung fibrosis, characterised by progressive and unrelenting ECM deposition, is a feature of multiple diseases (e.g. idiopathic pulmonary fibrosis (IPF), systemic sclerosis). Research on this topic commonly uses a bleomycin-induced mouse model. Peyser et al. isolated fibroblasts for single-cell RNA-seq in the early disease stage, and perhaps as expected, observed a significant increase in the proportion of cells in an “activate fibroblast” cluster [43•]. Importantly however, they showed that fibroblast number was not increased at this stage, fibroblast heterogeneity persisted in the fibrotic lungs and none of the signature genes in the activated cluster were exclusive. Notably, Acta2 (the gene encoding α-smooth-muscle-actin, αSMA) and TGFβ signalling-associated genes were only upregulated in a subset of “activated cells”, illustrating that an inclusive approach considering all of the parameters influencing their phenotype (Fig. 1a) is needed to capture the complexity of activated fibroblasts. A similar single-cell RNA-seq study identified an additional profibrotic population with high PDGFRβ expression [44]. Although PDGFRβ is known as a pericyte marker, trajectory analysis and direct comparisons of the transcriptional signatures suggest that lipofibrobasts are their major source.

Ageing seems to add another layer of complexity to lung fibrosis (Fig. 1b). Comparison of bleomycin-induced fibrosis in young and old mice revealed that with age, myofibroblasts acquire an apoptosis-resistant phenotype that is mediated by sustained activation of NADPH oxidase 4 (Nox4) leading to a redox imbalance and impaired induction of an Nrf2 anti-oxidative response [45]. Pharmacological Nox4 inhibition was able to resensitise myofibroblasts to apoptosis, thus reducing the duration of fibrosis in aged mice, and in turn prolonging their survival after lung injury. These findings could be extrapolated to human IPF tissue, which also showed a redox imbalance mediated by the same molecular mechanisms.

The cell composition in human lung fibrosis has been studied by comparing systemic sclerosis (SSc) lungs to healthy controls [42••]. This study detected a disease-associated, actively proliferating myofibroblast population that is thought to have undergone significant phenotypic changes during disease development, including upregulation of collagens and other profibrotic genes. Despite dramatic differentiation, the transcriptional signature was sufficiently consistent with only one of the two major cell populations in the healthy lung (MFAP5+) so that descent from this lineage could be predicted [42••]. Following a similar approach to dissect the stroma of human lung tumours at single-cell resolution, five distinct fibroblast subpopulations have been revealed with unique repertoires of ECM molecules [46]. It will be interesting to apply the proposed fibroblast stratification strategies (Fig. 1) to compare the cellular composition and relative fibroblast contributions of tumour-associated desmoplasia with other fibrotic lesions, in order to understand the influence of the cancer cell themselves and the special immune microenvironment.

### Liver

The fibroblasts of the liver, hepatic stellate cells (HSC) and portal fibroblasts, comprise approximately 15% of the total cells in the organ [47]. In homeostasis, HSCs are generally quiescent, residing in a special connective tissue space (space of Disse) between the sinusoids and the hepatocytes, where they play important roles in structural support and storing vitamin A. In contrast, portal fibroblasts do not store vitamin A lipids and are located around the bile duct in the portal tract, expressing characteristic markers including COL15A1, elastin and ectonucleoside triphosphate diphosphohydrolase-2 [13]. Both cell types can be activated by numerous physical and chemical insults and, when chronically activated, are key culprits of liver fibrosis. Notably, a lineage tracing study labelling myofibroblasts using *col1a1* promoter reported that HSCs were the major source of myofibroblasts (> 87%) in a chemical injury mouse model (carbon tetrachloride), whereas portal fibroblasts contributed predominantly to myofibroblasts (> 70%) in an early stage of cholestatic injury (bile duct ligation) [48].

Research of liver fibroblast heterogeneity is making significant headway into understanding how the different layers of complexity manifest in this organ system (Fig. 1a). For example, developmental HSC diversity was discovered by lineage-tracing Wilms’ Tumour 1 (WT1)-positive cells (which labels mesothelium) from development through to adulthood [49•]. This revealed two subpopulations of quiescent HSCs in healthy adult liver, one positive for the WT1 lineage and one negative, which have strikingly divergent transcriptomes and distinct contributions to liver fibrosis. Specifically, mesothelium-derived (WT1-lineage) cells were the major contributing cells to fibrotic lesions in numerous injury models. Interestingly, re-expression of WT1 in response to tissue damage (i.e. cells re-enacting their developmental gene expression signature) promoted cell de-differentiation/plasticity, and conversely, WT1 loss facilitated myofibroblast differentiation [49•].

Single-cell RNA-seq of adult mouse liver without stratifying on developmental origin provides a different perspective (Fig. 1b). Dobie et al. analysed PDGFRβ-positive cells (anticipated to label all mesenchymal cells) from healthy adult mouse liver and identified two mesenchymal populations (in addition to vascular smooth muscle cells), which they designated as HSCs (enriched for vitamin A–associated genes) and fibroblasts (enriched for ECM genes) [50•]. Interestingly, the authors also demonstrated regional heterogeneity of the HSCs, with transcriptomes varying with respect to proximity to the portal versus central vein. They identified that central vein–associated HSCs were the dominant pathogenic collagen-producing cells in a chemical injury model. Notably, these activated HSCs expressed high levels of lysophosphatidic acid receptor 1 (LPAR1), a G protein–coupled receptor that binds to lipid-signalling molecule LPA, and pharmacological inhibition significantly inhibited liver fibrosis. The site-specific features of the tissue influencing the cell phenotype (e.g. hypoxia [51]) will be interesting to understand (Fig. 1a) and may help to optimise anti-fibrotic therapies in the future. A similar single-cell RNA-seq study unveiled additional diversity within the activated HSC population [52]. Both an in vivo (carbon tetrachloride) and an in vitro (cultivation on plastic with serum) model resulted in four clusters of activated cells. S100A6 expression was shared across all populations, but the different groups varied in αSMA, collagens, immunological markers and stress-response genes, suggesting they may make unique contributions to fibrosis (as proposed in Fig. 1b) worth dissecting in the future.

A single-cell RNA-seq study of healthy versus cirrhotic human liver aiming to define alterations in niche components and signalling interactions in fibrotic disease uncovered four distinct mesenchymal clusters: vascular smooth muscle (MYH11), hepatic stellate (RGS5 high), mesothelial cells and SAMes (scar-associated mesothelial) cells [53••]. Notably, SAMes cells were expanded in cirrhotic livers and showed high expression of PDGFRα, fibrillar collagens and other pro-fibrotic genes, many of which are conserved from mouse [54]. Further clustering of SAMes cells revealed two subpopulations distinguished by OSR1 expression (odd-skipped related 1 transcription factor), which labelled periportal cells in healthy liver as well as cells in fibrotic lesions. This is additional evidence that, although they have some important distinct features, regional subpopulations (e.g. portal versus central) all have the potential to contribute to disease (Fig. 1b).

### Kidney

During kidney development in mouse and human, nephron patterning involves mesenchymal progenitor cell recruitment into the epithelial nephron precursor [55]. Lineage tracing experiments have shown that almost all kidney fibroblasts are derived from neural crest cells as they are lineage-labelled with myelin protein zero (P0) [56]. In adult homeostasis, fibroblasts reside in the renal interstitium and assume an interesting organ-specific function of erythropoietin expression. Resident fibroblasts also wrap around peritubular capillaries to provide stability to the vasculature, where they are thought to overlap considerably with pericytes. Upon acute kidney injury (AKI), fibroblasts detach from the capillaries and migrate to the site of damage, where they transdifferentiate to myofibroblasts, losing erythropoietin expression but gaining the ability to secrete scar-associated ECM (reviewed in [57]). If the damage is repeated or persistent, or particularly severe, AKI can progress to chronic kidney disease, with organ fibrosis as a hallmark of this scenario.

Although single-cell RNA-seq analysis has been performed on mouse and human kidney tissue in development and disease, generating a comprehensive cell atlas, the mesenchymal cell heterogeneity and in particular its functional significance in fibrosis remain poorly defined [58•, 59, 60••]. Stratifying the fibroblasts based on the four proposed variables and considering the dynamic changes in health and disease could help to provide more insights into these single-cell datasets (Fig. 1). 

Genetic lineage tracing experiments indicate that both resident fibroblasts and pericytes are precursors to the myofibroblasts that produce the bulk of the scar matrix. The picture is emerging that, beyond ECM deposition, resident fibroblasts display pro-inflammatory phenotypes upon tissue injury and even become inflammatory effector cells, by activating NF-κB signalling and secreting pro-inflammatory cytokines (reviewed in [61]). Curiously, with age, acute injury can lead to the development of tertiary lymphoid tissue within the kidney, suggesting age-dependent variations in the plasticity and fate of the pathological cell phenotype. Sato et al. discovered that only in aged mice fibroblasts could be stratified based on their localisation (i.e. within the tertiary lymphoid tissue versus in the surrounding areas) [62]. These two distributions were associated with different gene expression and function, with the surrounding cells expressing the machinery to produce retinoic acid, and the activated fibroblasts within the tertiary lymphoid tissue sustaining inflammation and impeding tissue repair. Interestingly, the fibroblast subset within the lymphoid territories expressed p75 neurotrophin receptor (NTR), a neural crest marker. Reinstatement of this marker that reflects the cells’ developmental origin is reminiscent of the WT1+ induction by activated HSCs in the liver [49•], which may indicate a de-differentiation process with an accompanying increase in plasticity. Indeed, these NTR+ cells are remarkably plastic, capable of maturing into CD21/CXCL13-positive follicular dendritic cells within this specialist tissue niche.

### Heart

Cardiac fibroblasts represent ~10–20% of all cardiac cells in adult mice and are distributed throughout the interstitial, perivascular and sub-epicardial spaces [63]. Similar to fibroblasts in other organs, it is very difficult to clearly define a fibroblast in the cardiac tissue because common markers such as CD90 (Thy1), FSP1 (fibroblast-specific protein 1) or vimentin are also expressed by many other cell types (reviewed in [64]). In mice, PDGFRα seems to be the most reliable pan-fibroblast marker in the adult heart. In homeostasis, cardiac fibroblasts have diverse functionality, providing important paracrine signals, ECM that has structural, mechanical, electrically insulating functions, and also responding in the event of tissue injury. Therefore, it is predictable that, amongst the fibroblasts that specialise to fulfil this range of tasks, there will be heterogeneous subsets defined by the four variables (Fig. 1a).

Developmentally, there are two main sources of fibroblasts that were revealed by lineage-tracing which somewhat segregate by location [65, 66]. Location within the heart is indeed expected to be an important regional variable strongly influencing (and reflecting) the resident fibroblasts; for example, the valves compared with the ventricular walls have remarkably different cellular composition, mechanical properties, metabolic demands and susceptibility to damage [15]. A single-cell RNA-seq analysis identified two major fibroblast populations in adult mouse ventricles; however, the distribution of expression of an epicardium marker amongst the cells indicated that these clusters do not reflect the two developmental origins [67•]. Similarly, a single-nucleus transcriptomic approach of early postnatal heart tissue identified two fibroblast populations that were actually only subtly different and both expressed high levels of specific ECM genes (periostin, fibrillin 1 and collagen 5a1) [68]. Further exploration into the overlap, sub-clustering and differing functions of these populations is warranted. Another single-cell RNA-seq analysis on all interstitial cell populations in the ventricles identified five relevant fibroblast cell populations in homeostasis: Sca1-low and Sca1-high, Wnt-expressing, myofibroblasts and activated fibroblasts [69••]. To better dissect the heterogeneity of these cells, the authors focussed on the PDGFRα-positive lineages. This actually exposed 11 populations, including subtypes expressing pro- and anti-fibrotic gene signatures, and two previously unrecognised groups, Wnt-expressing cells (which have enrichment of both positive and negative regulators of the pathway and are predicted to have anti-fibrotic and angiogenesis function) and transitory cells (predicted to be transitioning from Wnt-expressing to Sca1-low fibroblasts that have an intriguing secretory phenotype). Although more research on their distinct functionalities is required, some predictions on the roles of the 11 different populations could be inferred by the gene expression profiles and abundance at rest and following myocardial infarction (MI).

MI injury causes acute necrosis of cardiomyocytes, which then initiates a tissue repair response that inevitably forms a scar. Due to its lack of contractility and altered mechanical properties, the scar tissue severely compromises functionality of the organ. Although the scar is far from perfect, it is essential since fibroblast depletion using diphtheria toxin prior to MI injury leads to increased lethality because of inefficient collagen production and ventricular rupture [70]. It seems there is a balance to be struck between sufficient repair but minimised fibrosis; an understanding of the diverse fibroblasts that contribute to repair has the potential to uncover strategies to manipulate certain subsets to dampen the fibrotic response. Single-cell RNA-seq time-course experiments after MI injury in mice indicate that approximately 50% of cardiac fibroblasts become activated and start to express αSMA [69••]. It is thought that all cardiac fibroblast populations can contribute to the activated pool, and the scale suggests that it also extends beyond the infarcted area. Trajectory analysis of this time course of repair showed that the “activated” fibroblast population peaked in numbers at 3 days post-injury and could be split into three clusters: a non-proliferative, a cycling and a cluster transitioning between the two. The transcriptome of the non-proliferative but activated fibroblasts was most similar to the resting population, pointing to a gradual transition towards a myofibroblast fate. Fully differentiated myofibroblasts with stereotypical expression profiles including cell adhesion, ECM remodelling and angiogenesis genes dominated only at 7 days post-injury and also further divided into three obvious subpopulations. Two groups had high Tgfb1, Scx and Thbs4 expression, which are known drivers of cardiac fibrosis, but the third population expressed high level of anti-fibrosis genes including Wisp2, Sfrp2, Htra1 and Htra3, indicating that they will have contrasting functions in fibrosis. This is another prominent example of how fibroblast heterogeneity is able to dynamically change in disease conditions (Fig. 1b).

Although heart fibrosis is generally thought of as irreversible, some degree of resolution can be observed after acute injury and its subsequent repair process. In addition to apoptosis, lineage tracing experiments indicate that myofibroblasts can revert back to a less activated state [70] and acquire a new and stable differentiated status designated matrifibrocytes [71]. These express an ECM gene signature reminiscent of tendon, which is anticipated to promote a mature scar. Harnessing this cell plasticity may be therapeutically valuable.

## Conclusion and Future Outlook

Tissue analysis at a single-cell resolution has opened up incredible new insights into the cellular heterogeneity of different organs, and particularly an unexpected diversity amongst the fibroblasts. With sequencing costs decreasing and the sensitivity and sequencing depth increasing, we can expect numerous publications uncovering more and more subpopulations of fibroblasts in all tissues (e.g. including synovium or bone-marrow [72, 73]). New strategies to stratify fibroblasts that are guided by their functional diversity rather than the expression of specific marker genes are essential to understand and compare fibroblast heterogeneity in different organ systems and across species (Fig. 1).

We are only at the beginning of understanding the functional relevance of this heterogeneity for organ function in homeostasis and disease, and the challenges are numerous. Manipulating specific fibroblast types in vivo is technically challenging due to the lack of specific drivers, since so many of the genes expressed are overlapping. Also, although it may be possible to isolate the subpopulations using multiple markers, their phenotype may not be stable in culture conditions [23•, 74, 75]. Additional insight into subpopulation functionality may be gleaned from positional information, but this is lacking from most single-cell studies to date. Optimistically, new spatial transcriptomic technology is anticipated to bridge this knowledge gap [76, 77]. Indeed, the Human Cell Atlas initiative includes within their objectives the mapping of single cells within their tissue (https://www.humancellatlas.org/). Finally, translating the discoveries in animal models to the human setting is a difficult hurdle. Mouse work is impressively establishing the trajectory of certain cell types towards a pathological phenotype, but this is very difficult to replicate in human tissues, where we can typically only analyse the beginning (healthy tissue) and the end (advanced disease), and even that is usually with tissue from different individuals. However, a recent attempt to analyse the developmental trajectory of renal tumours in humans has produced promising results [60••].

Many of us hypothesise that there will be certain fibroblast subsets particularly responsible for driving fibrotic disease, and in turn believe that therapeutically manipulating the identity, behaviour or even survival of specific cells could be beneficial in treating these conditions. Therefore, motivation is high to push technical limits in order to understand the extrinsic and intrinsic mechanisms underlying the establishment and persistence of fibroblasts causative of pathological fibrosis.
